# Supplementary material for: A Simulation-Data-Based Machine Learning Model for Predicting Basic Parameter Settings of the Plasticizing Process in Injection Molding
Source: Polymers (Basel). 2021 Aug 10;13(16):2652. doi: 10.3390/polym13162652 (PMC8401074; doi:10.3390/polym13162652)
Supplement: Supplementary file 1 [file polymers-13-02652-s001.zip › polymers-1309672-supplementary.pdf]

Supplementary File

This file gives an overview of all the machine learning methods used in the paper. It shows all hyperparameters of the corresponding best model that was found.

All methods were implemented with Pythons library "sklearn".

Gaussian Process Regression:

Method explanation: 1.7. Gaussian Processes — scikit-learn 0.24.2 documentation

best model hyperparameters:

alpha=1e-05,

copy_X_train=True,

kernel=None,

n_restarts_optimizer=9,

normalize_y=False,

optimizer='fmin_l_bfgs_b',

random_state=None

Support Vector Regression:

Method explanation: sklearn.svm.SVR — scikit-learn 0.24.2 documentation

best model hyperparameters:

C=700,

cache_size=200,

coef0=0.0,

degree=3,

epsilon=0.1,

gamma='scale',

kernel='rbf',

max_iter=-1,

shrinking=True,

tol=0.001,

verbose=False

Polynomial Regression:

Method explanation: sklearn.preprocessing.PolynomialFeatures — scikit-learn 0.24.2 documentation

best model hyperparameters:

```
Pipeline(memory=None,
        steps=[('polynomial_features',
                PolynomialFeatures(degree=6, include_bias=False,
                                   interaction_only=False, order='C')),
               ('linear_regression',
                LinearRegression(copy_X=True, fit_intercept=True, n_jobs=None,
                                 normalize=False))],
        verbose=False)
```

Random Forest:

Method explanation: sklearn.ensemble.RandomForestRegressor — scikit-learn 0.24.2 documentation

best model hyperparameters:

bootstrap=True,

ccp_alpha=0.0,

criterion='mse',

max_depth=None,

max_features='auto',

max_leaf_nodes=None,

max_samples=None,

min_impurity_decrease=0.0,

min_impurity_split=None,

min_samples_leaf=1,

min_samples_split=2,

min_weight_fraction_leaf=0.0,

n_estimators=125,

n_jobs=None,

oob_score=False,

random_state=12,

verbose=0,

warm_start=False)

Gradient Boosting:

Method explanation: [sklearn.ensemble.GradientBoostingRegressor — scikit-learn 0.24.2 documentation]

best model hyperparameters:

alpha=0.9,

ccp_alpha=0.0,

criterion='friedman_mse',

init=None,

learning_rate=0.1,

loss='ls',

max_depth=3,

max_features=None,

max_leaf_nodes=None,

min_impurity_decrease=0.0,

min_impurity_split=None,

min_samples_leaf=1,

min_samples_split=2,

min_weight_fraction_leaf=0.0,

n_estimators=75,

n_iter_no_change=None,

presort='deprecated',

random_state=None,

subsample=1.0,

tol=0.0001,

validation_fraction=0.1,

verbose=0, warm_start=False)
